# Modulation of sulfur metabolism enables efficient glucosinolate engineering

**DOI:** 10.1186/1472-6750-11-12

**Published:** 2011-01-31

**Authors:** Morten E Møldrup, Fernando Geu-Flores, Carl E Olsen, Barbara A Halkier

**Affiliations:** 1 Department of Plant Biology and Biotechnology, Faculty of Life Sciences, University of Copenhagen, Thorvaldsensvej 40, DK-1871 Frederiksberg C, Denmark; 2 VKR Research Centre for Pro-Active Plants, Faculty of Life Sciences, University of Copenhagen, Thorvaldsensvej 40, DK-1871 Frederiksberg C, Denmark; 3 Department of Natural Sciences, Faculty of Life Sciences, University of Copenhagen, Thorvaldsensvej 40, DK-1871 Frederiksberg C, Denmark

## Abstract

**Background:**

Metabolic engineering in heterologous organisms is an attractive approach to achieve efficient production of valuable natural products. Glucosinolates represent a good example of such compounds as they are thought to be the cancer-preventive agents in cruciferous plants. We have recently demonstrated that it is feasible to engineer benzylglucosinolate (BGLS) in the non-cruciferous plant *Nicotiana benthamiana *by transient expression of five genes from *Arabidopsis thaliana*. In the same study, we showed that co-expression of a sixth *Arabidopsis *gene, *γ-glutamyl peptidase 1 *(*GGP1*), resolved a metabolic bottleneck, thereby increasing BGLS accumulation. However, the accumulation did not reach the expected levels, leaving room for further optimization.

**Results:**

To optimize heterologous glucosinolate production, we have in this study performed a comparative metabolite analysis of BGLS-producing *N. benthamiana *leaves in the presence or absence of *GGP1*. The analysis revealed that the increased BGLS levels in the presence of *GGP1 *were accompanied by a high accumulation of the last intermediate, desulfoBGLS, and a derivative thereof. This evidenced a bottleneck in the last step of the pathway, the transfer of sulfate from 3'-phosphoadenosine-5'-phosphosulfate (PAPS) to desulfoBGLS by the sulfotransferase AtSOT16. While substitution of AtSOT16 with alternative sulfotransferases did not alleviate the bottleneck, experiments with the three genes involved in the formation and recycling of PAPS showed that co-expression of *adenosine 5'-phosphosulfate kinase 2 *(*APK2*) alone reduced the accumulation of desulfoBGLS and its derivative by more than 98% and increased BGLS accumulation 16-fold.

**Conclusion:**

Adjusting sulfur metabolism by directing sulfur from primary to secondary metabolism leads to a remarkable improvement in BGLS accumulation and thereby represents an important step towards a clean and efficient production of glucosinolates in heterologous hosts. Our study emphasizes the importance of considering co-substrates and their biological nature in metabolic engineering projects.

## Background

The plant kingdom is an extensive source of valuable compounds with a wide range of applications, most notably, in medicine. However, the availability of phytochemicals in their natural sources is often limited. In recent years, the efficient production of bioactive plant natural products has mainly been attempted through the metabolic engineering of microorganisms, as exemplified by the successful production of artemisinic acid in yeast [1]. Although microorganisms are generally considered easier to engineer, plants themselves present a series of advantages as production organisms. For example, they only require soil, water, and sun light for their growth (which is unarguably CO_2 _friendly), and their care does not need highly educated personnel or specialized equipment. Therefore, there is a growing interest in the metabolic engineering of plants for the production of high-value bioactive compounds. Still, pathway engineering in plants is in its infancy and improvements are needed to reach the same level of flux control and yield optimization as seen in microorganisms. Part of the explanation for this is that stable plant transformations are notoriously time-consuming, making the challenge of stably engineering a whole biosynthetic pathway - without prior confirmation of feasibility - an endeavour beyond the scope of most research projects.

To obtain fast answers about the feasibility of engineering projects, we have turned to an established protocol for transient transformation of *Nicotiana benthamiana *[2], which has allowed us to rapidly probe the engineering of the sulfur-rich phytochemicals called glucosinolates (GLS). Apart from being well known as biopesticides, glucosinolates are thought to be the cancer-preventive agents in cruciferous plants [3]. Recently, we demonstrated the production of benzylglucosinolate (BGLS) in leaves of *N. benthamiana *by transient co-expression of five known biosynthetic genes from *Arabidopsis thaliana *[4]. The accumulation of BGLS was, however, concomitant with a disproportionately higher accumulation of a putative intermediate, the glutathione conjugate GS-B (~100-fold higher at 6 dpi). This problem was solved by the discovery of γ-glutamylpeptidase 1 (GGP1), which was able to cleave GS-B *in vitro*, and whose presence in BGLS-producing *N. benthamiana *leaves led to a strong reduction in GS-B accumulation (> 99% at 6 dpi) and a substantial increase in BGLS levels (~4-fold at 6 dpi) [4]. The discovery of GGP1 showed that the transient system was not only useful for assessing the feasibility of engineering a given pathway, but also for gene discovery.

Although the presence of GGP1 in BGLS-producing *N. benthamiana *leaves resolved a major metabolic bottleneck, the increase in accumulation of BGLS did not account for the reduction in GS-B accumulation. In fact, only a small portion of the cleaved GS-B was converted to BGLS [4]. This suggested that an additional downstream bottleneck was present, and that BGLS production in this system could be further improved. In the present study, we report the identification of this bottleneck and its alleviation, which led to greatly increased BGLS levels. Our results are discussed in terms of the heterologous production of glucosinolates and in the general context of pathway engineering.

## Methods

### Generation of plant expression constructs

All constructs were assembled in *Escherichia coli *strain DH10B by USER cloning of coding sequences (CDSs) into the plasmid pCAMBIA3300-35Su [5]. The CDS of *SAL1 *(At5g63980) was amplified by PCR from cDNA made from leaves of *A. thaliana *Col-0. The CDSs of the remaining genes were amplified from existing cDNA clones: *APK2 *(At4g39940) from ABRC clone u21470; *ATPS1 *(At3g22890) from ABRC clone u10843; *UGT74B1-SUR1 *(At1g24100-At2g20610) from ORF2nat [6]; and finally, *AtSOT16 *(At1g74100), *AtSOT17 *(At1g18590), *AtSOT18 *(Ag1g74090), and *PAPS-S *from published cDNA clones [7,8]. *AtSOT16 *has been previously referred to by us as *AtST5a *[4,6].

### Transient co-expression in leaves of N. benthamiana

Transient transformation of leaves of *N. benthamiana *was performed using *Agrobacterium tumefaciens *strain GV3850 and the silencing suppressor p19 [2]. Co-transformations were done by mixing different *Agrobacterium *strains (each carrying a single expression construct) in equal volumes prior to infiltration (Total OD_600 _of the mixed strains was 0.5), as previously described [4]. When comparing the effects of unequal number of strains, a strain transformed with an expression plasmid encoding for GFP [2] was included so as to obtain equal individual ODs across different strain combinations.

### GLS and LC-MS analysis

Leaves were harvested at 6 dpi by cutting four leaf discs from the *Agrobacterium*-infiltrated area and homogenizing them in 400 μl of MeOH containing 0.02 mM sinigrin as internal standard. Glucosinolates were quantified by HPLC-UV using the desulfoglucosinolate method [9] and BGLS amounts were calculated relative to sinigrin using a relative response factor of 0.8 [10]. Metabolite analysis was performed by LC-MS as previously described [4], and exact masses were obtained using a micrOTOF-Q detector (Bruker Daltonics).

### Endogenous N. benthamiana sulfatase activity assay

*N. benthamiana *leaves infiltrated with *Agrobacterium *harboring *AtSOT16 *were harvested at 6 dpi and homogenized in protein extraction buffer [250 mM sucrose, 100 mM Tris-HCl pH 7,5, 50 mM NaCl, 2 mM EDTA, 5% PVPP, 5 mM DTT and 1X 'Complete Protease Inhibitor' (Roche Molecular Biochemicals)]. After centrifugation at 20 000 *g *at 4ºC for 20 min, 50 μg of soluble protein (from the supernatant) was added to 200 μl reaction buffer I [100 mM Tris-HCl pH 8.0, 10 mM MgCl_2_, 1 mM PAP (3'-phosphoadenosine 5'-phosphate, Sigma-Aldrich) and 1 mM BGLS (Calbiochem)]. The reaction mixtures were incubated at 25ºC for one hour and stopped and extracted by addition of 400 μl ethyl acetate. A fraction of the ethyl acetate phase was evaporated and resuspended in acetonitrile. Sulfatase activity, represented by the formation of desulfoBGLS, was quantified by HPLC-UV [9].

As a positive control for the extraction of active soluble proteins, a sulfotransferase assay was carried out in parallel by adding 50 μg protein from 20 000 *g *supernatant to 100 μl of reaction buffer II [100 mM Tris-HCl pH 8.0, 10 mM MgCl_2_, 0.1 mM PAPS (3'-phosphoadenosine 5'-phosphosulfate, Calbiochem) and 0.1 mM dBGLS (obtained by desulfation of BGLS)]. The assay mixtures were incubated at 25ºC for one hour and stopped by addition of 400 μl of methanol. Sulfotransferase activity, represented by the formation of BGLS, was quantified by HPLC-UV using the desulfoglucosinolate method [9].

## Results

### Metabolite analysis of BGLS-producing *N. benthamiana*

**Figure 1:**
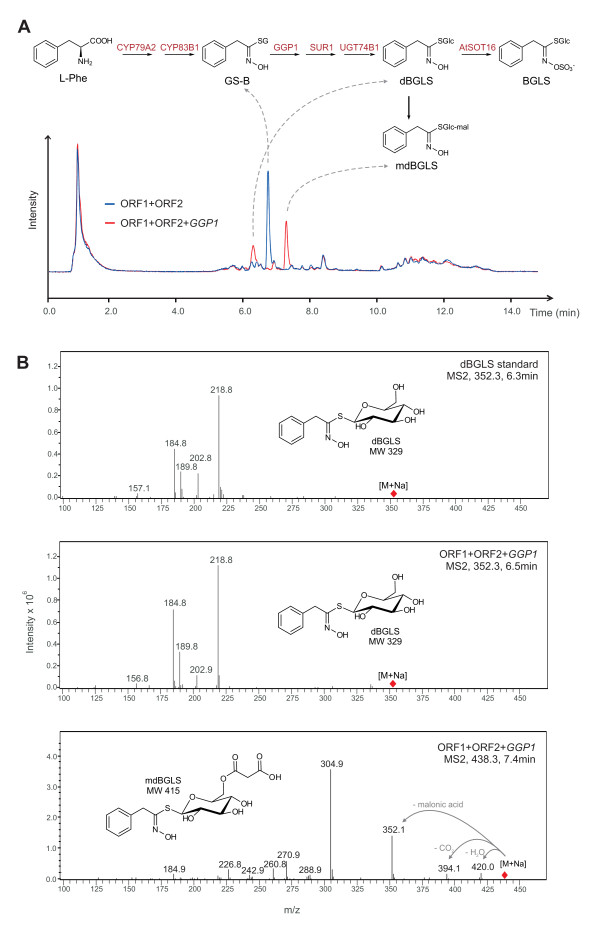
**Metabolite analysis of BGLS-producing *N. benthamiana *leaves**. A) Total ion chromatograms from the LC-MS analysis of *N. benthamiana *leaves expressing ORF1 (coding for CYP79A2 and CYP83B1) and ORF2 (coding for SUR1, UGT74B1, and AtSOT16), in the presence or absence of *GGP1 *(red and blue traces, respectively). A scheme of the engineered pathway is inserted, where the identities of the three main chromatogram peaks have been assigned to by dashed arrows. Metabolite abbreviations are written in black, and enzyme names are written in red. B) MS2 fragmentation patterns of the [M+Na]^+ ^adducts of standard dBGLS (upper panel) and of the prominent compounds eluting at 6.5 min (middle panel) and 7.4 min (lower panel) in the analysis of the extracts of *N. benthamiana *leaves expressing ORF1, ORF2, and *GGP1*. The chemical structures of the proposed compounds are inserted. For mdBGLS, the location of the malonyl residue at position 6 is only tentative.

We performed LC-MS analyses of extracts from *Agrobacterium*-infiltrated *N. benthamiana *leaves producing BGLS. The analyses confirmed that, when GGP1 is present together with the rest of the BGLS enzymes (encoded by the 2A-polycistronic constructs ORF1 and ORF2) [4], the peak of the glutathione conjugate GS-B was almost completely eliminated (Figure 1A). However, two major peaks appeared instead (Figure 1A). Based on MS2 fragmentation patterns (Figure 1B) and exact mass determination (4-digit mass errors of < 1.6 ppm), these peaks were annotated as desulfoBGLS (dBGLS) and malonylated dBGLS (mdBGLS). The identity of the dBGLS peak was confirmed by comparison to an authentic standard (Figure 1B). Mean dBGLS content at 6 dpi was quantified to be 1.58 ± 0.16 nmol/mg fresh weight (nmol/mg fw), which is ~five-fold higher than the corresponding BGLS concentration. The size of the mdBGLS peak was comparable to that of dBGLS (Figure 1A), but the lack of a standard prevented its quantification.

Malonylation of metabolites and xenobiotics is a general plant storage and detoxification reaction, and unspecific malonyltransferases have been previously characterized in *Nicotiana tabacum *[11]. The co-occurrence of dBGLS and mdBGLS could thus readily be explained as a consequence of malonylation of the accumulating dBGLS by an unspecific endogenous malonyltransferase. dBGLS itself is the last intermediate of the BGLS pathway (Figure 1A), hence the accumulation of both dBGLS and mdBGLS suggested that the last reaction of the pathway, i.e. incorporation of sulfate by the sulfotransferase AtSOT16, constituted a metabolic bottleneck. Alternatively, the presence of dBGLS in BGLS-producing *N. benthamiana *could be due to the desulfation of intact BGLS by an unspecific endogenous sulfatase. However, when we tested soluble protein extracts from *N. benthamiana *leaves for *in vitro *sulfatase activity against BGLS, we were not able to detect any dBGLS (data not shown). Therefore, we focused on the sulfotransferase reaction in the remaining experiments.

### Testing of alternative *Arabidopsis *desulfoglucosinolate sulfotransferases

*A. thaliana *ecotype Col-0 has three desulfoglucosinolate sulfotransferases: AtSOT16, AtSOT17 and AtSOT18, all of which were reported to convert dBGLS into BGLS *in vitro *[7,12]. The sulfotransferase that we had previously chosen to be part of the 2A multicistronic construct ORF2 was AtSOT16, which has the highest *k*_cat_/*K*_m _towards dBGLS [12]. However, since the preferred *in vitro *substrates do not always correspond to the preferred *in vivo *substrates, we tested the ability of AtSOT17 and AtSOT18 to support BGLS engineering. Moreover, the AtSOT16 derived from ORF2 carried an N-terminal His-tag as well as 17 extra amino acids (from the 2A sequence) in its C-terminus [6]. As these additional amino acids might interfere with catalytic functions, we also tested a native version of AtSOT16 in parallel with native AtSOT17 and AtSOT18.

**Figure 2:**
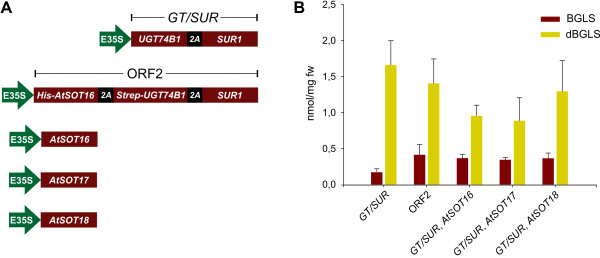
**AtSOT-dependent BGLS and dBGLS accumulation**. A) Expression constructs used for testing different versions of AtSOTs. *His *B) BGLS and dBGLS accumulation in leaves of *N. benthamiana *expressing ORF1 and *GGP1 *in combination with the indicated constructs at 6 dpi. Each data point represents the mean of eight biological replicates. Error bars represent standard deviations.

In order to have an AtSOT16-free version of ORF2, we generated the expression construct *GT/SUR*, which encoded only the glucosyltransferase UGT74B1 and the *C-S *lyase SUR1 (Figure 2A). In addition, three expression constructs encoding for, respectively, native AtSOT16, AtSOT17 and AtSOT18 were generated (Figure 2A) and individually co-infiltrated with ORF1 (coding for CYP79A2 and CYP83B1), *GGP1 *and *GT/SUR*. We subsequently compared BGLS and dBGLS accumulation in leaves infiltrated with the different strain combinations. No difference was observed in BGLS accumulation, neither between the tagged (from ORF2) and the un-tagged AtSOT16, nor between the three AtSOT isoforms (Figure 2B). The level of dBGLS remained slightly less constant, but more than twice as high as the BGLS content in all combinations (Figure 2B), which demonstrated that the alternative sulfotransferases could not alleviate the metabolic bottleneck.

The negative control without any AtSOT gave only a ~50% reduction in BGLS production when compared to all other combinations (Figure 2B). Though reduced, the production of 0.17 ± 0.04 nmol BGLS/mg fw in the absence of an *Arabidopsis *sulfotransferase shows that endogenous *N. benthamiana *sulfotransferases were able to catalyze the reaction. This is in agreement with a previous result, where we showed that, upon *in vivo *feeding of phenylacetothiohydroximate (the penultimate intermediate in the BGLS pathway) to wildtype *Nicotiana tabacum*, BGLS was produced, thereby demonstrating that the two last steps in the pathway could be catalyzed in a *Nicotiana *species by endogenous glucosyltransferases and sulfotransferases [6]. This results supported the hypothesis that the late enzymes of glucosinolate biosynthesis were recruited from general detoxification pathways. Nevertheless, and similarly to the present sulfotransferase experiment in *N. benthamiana*, the presence of an AtSOT did improve the accumulation of BGLS significantly [6].

### PAPS as limiting co-substrate

All known sulfotransferases utilize 3'-phosphoadenosine-5'-phosphosulfate (PAPS) as the activated form of sulfate, and the last step of the glucosinolate pathway is no exception. Furthermore, sulfotransferase reactions not only produce the sulfated metabolite, but have also adenosine-3',5'-bisphosphate (PAP) as a by-product.

**Figure 3:**
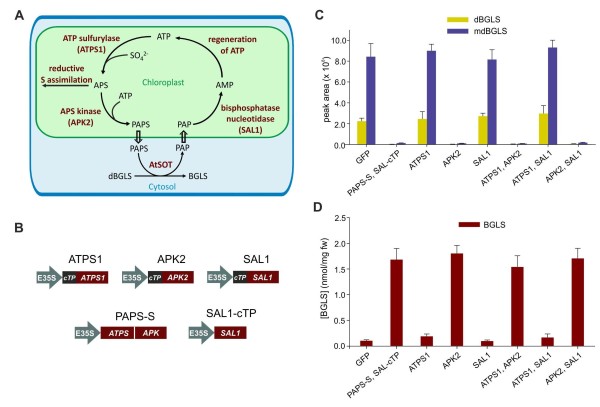
**The effect of PAPS formation and recycling genes on BGLS accumulation in *N. benthamiana***. A) Scheme of the link between formation and recycling of PAPS and the sulfotransferase reaction converting dBGLS to BGLS. Metabolites names are written in black, enzyme names are written in red, and the particular *Arabidopsis *isoforms used in this study are presented in parenthesis. Line arrows denote chemical conversions, while block arrows denote transport steps. B) Expression constructs coding for different PAPS formation and recycling enzymes. The term '*cTP*' refers to a native CDS fragment that encodes a chloroplast targeting peptide. C) dBGLS and mdBGLS accumulation in *N. benthamiana *leaves expressing ORF1, ORF2, and *GGP1 *in combination with the indicated constructs at 6 dpi. Metabolite concentrations are expressed as peak areas from extracted ion chromatograms. D) BGLS accumulation in *N. benthamiana *leaves expressing ORF1, ORF2, and *GGP1 *in combination with the indicated constructs at 6 dpi. For both C) and D), each data point represents the mean of four biological replicates, and error bars represent standard deviations.

PAPS is biosynthesized from adenosine-5'-triphosphate (ATP) and inorganic sulphate (SO_4_^2-^) in two enzymatic steps. First, ATP sulfurylase (ATPS) sulfates ATP to form APS (adenosine 5'-phosphosulfate). Second, APS kinase (APK) phophorylates APS to form PAPS. The phosphate donor for this reaction is another molecule of ATP (Figure 3A) [13]. APK is located at an important metabolic branchpoint in sulfur assimilation, because a competing reaction, catalyzed by APS reductase, channels APS into the reductive assimilatory pathway leading to cysteine and, further downstream, to glutathione (Figure 3A). After sulfation, the by-product PAP is hydrolysed to AMP (adenosine-5'-monohosphate) by a bisphosphate nucleotidase. This reaction is biologically important not only because it removes PAP, which is an inhibitor of sulfotransferase reactions [12], but also because AMP can then be regenerated to form ATP, leading to actual recycling of the adenosine moiety of PAPS (Figure 3A). In *Arabidopsis*, the hydrolysis of PAP is most likely catalyzed by the multifunctional protein SAL1 [14].

*In silico *microarray-based co-expression databases such as ATTED-II [15] and CressExpress [16] are powerful tools for elucidating metabolic networks in *Arabidopsis*. We have previously used such databases to identify genes in the GLS pathway [4,9]. In ATTED-II, searches were performed using both *SUR1 *and *CYP83B1 *as query. *ATP sulfurylase 1 *(*ATPS1*), *APS kinase 1 *(*APK1*), *APS kinase 2 *(*APK2*) and *SAL1*, all of which are likely to be involved in the formation and recycling of PAPS, were among the top 24 co-expressed genes for both queries, in addition to many known GLS biosynthetic genes (data not shown). A similar picture was seen in CressExpress, where *APK2, APK1 *and *ATPS1 *were among the top five co-regulated genes when five GLS biosynthetic genes (*CYP79B2*, *CYP83B1*, *SUR1*, *UGT74B1*, and *AtSOT16*) were used as query (data not shown). Therefore, we hypothesized that the lack of a highly efficient PAPS formation and recycling machinery in *N. benthamiana *prevented the sulfotransferase reaction from being carried to completion.

A relationship between APK genes and GLS biosynthesis was recently found by Mugford *et al.*, who demonstrated that the *Arabidopsis apk1/apk2 *double knockout mutant had reduced levels of GLS and accumulated desulfoglucosinolates [17]. Furthermore, it has been shown that *ATPS1, ATPS3, APK1*, and *APK2 *are regulated by MYB transcription factors known to regulate both aliphatic and indolic glucosinolate biosynthesis [18]. This further suggested that the formation of PAPS could be limiting in BGLS-producing *N. benthamiana *leaves.

To test the hypothesis that an increased PAPS formation and recycling would aid BGLS production in leaves of *N. benthamiana*, we generated individual plant expression constructs carrying the native coding sequences of ATPS1, APK2 and SAL1, respectively. However, these three proteins carry predicted chloroplast targeting peptides (cTPs) and their fusions to fluorescent proteins have been shown to localize to chloroplasts [17,19,20], whereas the glucosinolate pathway is proposed to be cytosolic [21]. This may pose a problem, as the co-substrate PAPS would have to be transported very efficiently from the chloroplast to the cytosol, while the by-product PAP would have to be mobilized efficiently in the opposite way. As a means to overcome this potential problem, we tested in parallel whether the PAPS formation and recycling machinery could be established in the cytosol. This was attempted using an ATPS-APK fusion protein from the marine worm *Urechis caupo *lacking a cTP, also referred to as PAPS synthetase (PAPS-S) [8], and a truncated version of SAL1 without its native cTP, which we named SAL1-cTP.

*Agrobacterium *strains harbouring the different PAPS formation and recycling genes (Figure 3B) were infiltrated into *N. benthamiana *leaves together with strains harbouring the BGLS biosynthetic genes, both individually and in selected combinations. In all experiments where APK2 was included, the levels of dBGLS and mdBGLS were reduced > 98% (Figure 3C). This was accompanied by a ~16-fold increase in mean BGLS levels (Figure 3D). ATPS1 and SAL1 did not affect the content of BGLS, dBGLS or mdBGLS any further, indicating that the endogenous ATPS and bisphosphate nucleotidase activities sufficiently support a highly increased PAPS formation and recycling, and that only the APK activity was limiting. The accumulation of BGLS, dBGLS, and mdBGLS in the presence of PAPS-S and SAL1-cTP, both lacking cTPs, did not differ from the accumulation seen with the plastid localized APK2. This suggests that the shuttling of PAPS and PAP across chloroplast membranes was not a limiting factor. In the presence of APK2, mean BGLS levels reached 1.80 ± 0.16 nmol/mg fw at 6 dpi. This is equivalent to the total GLS levels in rosette leaves of *Arabidopsis *Col-0 before bolting [22].

## Discussion

In this study, we have successfully optimized the heterologous production of BGLS in leaves of *N. benthamiana*, reaching glucosinolate levels that are equal or higher than those observed in plants that produce glucosinolates naturally. The optimization was enabled by the identification of a bottleneck in the last biosynthetic reaction, the transfer of sulfate to dBGLS by a sulfotransferase. The bottleneck was resolved solely by co-production of APK2, an enzyme that converts the intermediate APS in the sulfur assimilatory pathway into the sulfotransferase co-substrate, PAPS.

In mammalian systems, where numerous xenobiotics follow sulfation-dependet detoxification routes, sulfate transfer has been described as a high-affinity, low-capacity process [23]. The identification of the sulfotransferase bottleneck in our engineered BGLS pathway indicates that sulfate transfer is also a low-capacity process in non-cruciferous plants. However, the exact reasons for the low capacity are different. In rats and mice, the limiting factors appear to be the availability of sulfate and the sulfotransferases themselves [23]. Because the sulfotransferase problem in *N. benthamiana *was completely solved by co-expressing *APK2*, the provision of PAPS was clearly the limiting factor (and not sulfate availability nor the sulfotransferase AtSOT16).

Even under PAPS-limiting conditions, the addition of either AtSOT resulted in increased BGLS accumulation as compared when only endogenous *N. benthamiana *sulfotransferases were available. This increase can be speculated to be even more pronounced under PAPS unlimited conditions (i.e. in the presence of *APK2*). Conversely, it was not possible to determine any difference in the performance of the three AtSOTs under PAPS-limiting conditions, and future studies could therefore focus on determining the optimal AtSOT for production of specific GLS in the presence of *APK2*.

The finding that *APK2 *alone could resolve the sulfotransferase bottleneck also demonstrates that other PAPS-related activities, namely ATPS and bisphosphate nucleotidase activities were not limiting either. This likely reflects that the two latter enzymes participate in important PAPS-independent pathways. ATPS synthesizes the central sulfur assimilation intermediate APS, which can either be used in the reductive sulfur assimilation pathway leading to cysteine and glutathione or converted into to PAPS by an APK. In turn, bisphosphate nucleotidases not only hydrolyze PAP, but also inositol polyphosphate [14] and have been linked to numerous physiological processes such as salt, cold, and drought tolerance [24], RNA silencing [25], and leaf morphogenesis [26].

Although usually drawn as a linear pathway, the glucosinolate pathway is fueled by the co-substrates NADPH, glutathione, UDP-glucose, and PAPS [21]. While the three former co-substrates are used in many ubiquitous processes in primary metabolism, most sulfation processes are considered part of secondary metabolism [17]. Therefore, the formation and regeneration of NADPH, glutathione, and UDP-glucose is expected to be under more stringent control than that of PAPS (for example, by strict feedback regulation). This helps rationalizing why efficient glucosinolate production in *N. benthamiana *does not seem to require the engineering of co-substrates other than PAPS.

## Conclusion

Our study shows that modulation of sulfur metabolism towards enhanced PAPS biosynthesis enables an efficient heterologous production of glucosinolates in *N. benthamiana*. This represents an important step towards a clean and efficient production of glucosinolates in heterologous hosts and emphasizes the importance of considering co-substrates and their biological nature in metabolic engineering projects.

## Abbreviations

BGLS: benzylglucosinolate; dBGLS: desulfobenzylglucosinolate; mdBGLS: malonyldesulfobenzylglucosinolate; PAPS,: 3'-phosphoadenosine 5'-phosphosulfate; PAP: 3'-phosphoadenosine 5'-phosphate; APS: adenosine 5'-phosphosulfate; ATPS ATP: sulfurylase; APK, APS: kinase; PAPS-S, PAPS: synthetase.

## Authors' contributions

MEM participated in the design of the study, carried out most of the experimental work and drafted the manuscript. FG participated in the design of the study, helped carrying out the experimental work, and contributed to manuscript drafting and revising. CEO performed the LC-MS. BAH was overall study director and supervisor, participated in study design, helped to revise the manuscript and obtained the funding. All authors read and approved the final version.
